# T-bet Expression in Peripheral Th17.0 Cells Is Associated With Pulmonary Function Changes in Sarcoidosis

**DOI:** 10.3389/fimmu.2020.01129

**Published:** 2020-07-22

**Authors:** Nicholas K. Arger, Siddharth Machiraju, Isabel E. Allen, Prescott G. Woodruff, Laura L. Koth

**Affiliations:** ^1^Division of Pulmonary and Critical Care, University of California, San Francisco, San Francisco, CA, United States; ^2^Department of Epidemiology and Biostatistics, University of California, San Francisco, San Francisco, CA, United States

**Keywords:** sarcoidosis, Th17, Th17.1, Th1, T-bet, RORγt, interferon-gamma, chemokine

## Abstract

**Background:** Interferon-gamma (IFN-γ) is a key mediator of sarcoidosis-related granulomatous inflammation. Previous findings of IFN-γ-producing Th17 cells in bronchoalveolar lavage fluid from sarcoidosis patients invokes the transition of Th17.0 cells to Th17.1 cells in the disease's pathogenesis. Since the T-bet transcription factor is crucial for this transition, the goal of this study was to determine if T-bet expression in Th17.0 cells reflects the extent of granulomatous inflammation in sarcoidosis patients as assessed by clinical outcomes.

**Methods:** Using a case-control study design, we identified two groups of sarcoidosis subjects (total *N* = 43) with pulmonary function tests (PFTs) that either (1) changed (increased or decreased) longitudinally or (2) were stable. We used flow cytometry to measure the transcription factors T-bet and RORγt in Th1, Th17.0, and Th17.1 cell subsets defined by CCR6, CCR4 and CXCR3 in blood samples. We compared the percentages of T-bet^+^ cells in RORγt^+^Th17.0 cells (defined as CCR6^+^CCR4^+^CXCR3^−^) based on subjects' PFT group. We also assessed the relationship between the direction of change in PFTs with the changes in %T-bet^+^ frequencies using mixed effects modeling.

**Results:** We found that T-bet expression in subjects' RORγt^+^Th17.0 cells varied based on clinical outcome. The T-bet^+^ percentage of RORγt^+^Th17.0 cells was higher in the cases (subject group with PFT changes) as compared to controls (stable group) (27 vs. 16%, *p* = 0.0040). In comparisons before and after subjects' PFT changes, the T-bet^+^ frequency of RORγt^+^Th17.0 cells increased or decreased in the opposite direction of the PFT change. The percentage of these T-bet^+^ cells was also higher in those with greater numbers of involved organs. Serum levels of interferon-γ-induced chemokines, CXCL9, CXCL10, and CXCL11, and whole blood gene expression of IFN-γ-related genes including *GBP1, TAP1*, and *JAK2* were independently positively associated with the T-bet^+^ frequencies of RORγt^+^Th17.0 cells.

**Conclusions:** These data suggest that expression of T-bet in Th17.0 cells could reflect the extent of granulomatous inflammation in sarcoidosis patients because they represent a transition state leading to the Th17.1 cell phenotype. These findings indicate that Th17 plasticity may be part of the disease paradigm.

## Introduction

Sarcoidosis is a systemic disease in which granulomatous inflammation affects the lungs in the vast majority of patients (1). Its etiology remains unknown and no cure currently exists. Multiple studies have established the importance of IFN-γ-producing CD4^+^ T helper (Th) cells in sarcoidosis, which have been traditionally designated as “Th1” cells (2–6). In recent years, additional Th cell populations have been recognized in sarcoidosis, including IL-17-producing “Th17” cells (7–10). We previously identified two different CD4^+^ Th17 subsets in sarcoidosis patients using the expression of chemokine receptors CCR6, CCR4, and CXCR3 (11). In blood, we observed Th17.0 (CCR6^+^CCR4^+^CXCR3^−^) cells with increased frequencies in sarcoidosis compared to health. The majority of these cells produced IL-17 after *ex vivo* stimulation. In contrast, in bronchoalveolar lavage (BAL), we observed “Th17.1” (CCR6^+^CCR4^−^CXCR3^+^) cells, with increased frequencies in sarcoidosis compared to health (11, 12). We found that the majority of Th17.1 cells produced IFN-γ while only a small fraction produced IL-17 upon *ex vivo* stimulation (11). The increased proportion of Th17.0 cells in the circulation accompanied by an increased proportion of Th17.1 cells in the BAL led us to consider whether these findings might be the result of Th17 plasticity, whereby circulating Th17.0 effector cells polarize into Th17.1 cells and accumulate in the lung tissue where the granulomatous inflammation is located.

Prior studies have elucidated how Th17.0 cells can polarize or transition into Th17.1 cells. The initial polarization of Th17.0 effector cells from naïve T cells occurs under the control of the orphan nuclear hormone receptor RORγt (13–17). During this polarization, the chemokine receptors CCR6 and CCR4 are upregulated (18–20). In this context, the transcription factor RORγt is used to define Th17.0 cells (15–17). The mechanism by which Th17.0 cells “polarize” into Th17.1 cells has been elucidated through *in vitro* stimulation with IL-12 and IFN-γ. This stimulation causes upregulation of the transcription factor T-bet (21–24). T-bet is the main transcription factor that controls polarization of naïve T cells to Th1 cells (25–29). Once T-bet is activated, several downstream genes are upregulated including those for CXCR3 and IFN-γ (26–28). Based on this collective T cell biology, we speculate that T-bet upregulation in Th17.0 cells in sarcoidosis patients may be initiated by exposure to IL-12 and IFN-γ in lymph nodes or tissues containing granulomatous inflammation (such as the lung). This Th17 plasticity allows them to express both RORγt and T-bet transcription factors and as well as pathogenic cytokines (IFN-γ) and the complement of chemokine receptors including CXCR3 that permit homing from blood to sites of inflammation such as the lung (30). In our study, we hypothesized that the expression of T-bet in circulating Th17.0 cells prior to upregulation of CXCR3 may serve as an indirect measure of the extent of interferon-driven inflammation to which the Th17.0 cells are exposed. To test this, we used flow cytometry to compare the T-bet-expressing frequencies in peripheral blood Th17.0 (RORγt^+^CCR6^+^CCR4^+^CXCR3^−^) cells between sarcoidosis subjects with different clinical trajectories defined by longitudinal changes in lung function and immunosuppression use.

## Materials and Methods

### Clinical Cohort

We enrolled subjects who met diagnostic criteria for sarcoidosis per guidelines endorsed by the American Thoracic Society (31) as previously described (32). The study design for this cohort did not require individuals to be newly diagnosed to participate. Follow-up visits were performed every 6–12 months for up to 66 months (~5 years). At each visit, we performed blood sampling and collected clinical data. These data included: demographics (age, sex, and self-identified race), chest radiography at initial visit, organ involvement at the initial visit (as assessed by physician review of medical records) (32); and pulmonary function tests (PFTs), which included forced expiratory volume in 1 second (FEV1) percent predicted (%pred), forced vital capacity (FVC %pred), diffusing capacity for carbon monoxide (DLCO %pred), and total lung capacity (TLC %pred). We obtained immunosuppression use history, including dosages of oral corticosteroids or disease-modifying antirheumatic drugs (DMARDs), specifically, methotrexate, azathioprine, mycophenolate, colchicine, hydroxychloroquine, or anti-TNF-α therapy that subjects were actively taking at the time of their study visits. For the current analysis, we also included serum protein (chemokine levels for CXCL9, CXCL10, and CXCL11) and whole blood RNA transcript levels of IFN-γ-related genes that were measured in the same blood samples as previously described (33–35).

### Selection of Study Subjects

We used a case-control study design with criteria based on (1) lung function changes and (2) immunosuppressive treatment history to compare cell populations between sarcoidosis subjects with different clinical trajectories. Cases were defined by having a change in absolute FVC or DLCO of 10% or 15% (33, 36, 37), respectively, between any two visits separated by 6 months or greater. Cases could have been on or off treatment at the time of the blood draw (Figure S1). For all the cases, we identified the two visits between which the PFT change occurred. We then analyzed blood samples from the first of these two visits (before their lung function change occurred). In a subset of cases, we also analyzed blood samples from the second of these two visits (at the visit in which the change in lung function was measured). This subset of subjects with two measurements included subjects not on immunosuppression with a PFT decline (to avoid the effect of immunosuppression on T cell function) and all subjects with a PFT increase, some of whom were on immunosuppression (since the sample size was small for this group). We used a change in lung function as a surrogate of on-going granulomatous inflammation or “disease activity” for two main reasons: (1) there is a lack of reliable and accurate non-invasive tests for active granulomatous inflammation and (2) we did not have longitudinal radiographic imaging at each follow up visit, which also can be used to infer disease activity. For controls, we used criteria of (1) no change in FVC and DLCO measurements for at least 24 months following the enrollment blood draw used in this analysis and (2) no use of immunosuppression prior to or during the study period. Of note, we did not include FEV1 in our criteria for PFT changes due to the fact that other processes such airways disease independent of sarcoidosis could affect this value over time; plethysmography was not performed at every visit, therefore TLC was also not used as a criterion.

### Flow Cytometry

We employed a 12-parameter flow cytometry panel using an LSRII cytometer (BD Biosciences, San Jose, CA) in the UCSF Flow Cytometry Core (www.flow.ucsf.edu). Samples were blinded and randomized to 8 different batches with cases and controls distributed to each batch. Cases with longitudinal samples were analyzed in the same batch. At time of sample collection, we isolated peripheral blood mononuclear cells (PBMCs) using Leucosep^TM^ tubes then froze them in FBS with 10% DMSO for storage in our liquid nitrogen biorepository. For each batch, samples were thawed in RPMI media, counted, and stained on ice in the dark. Details for antibody staining reagents are listed in Table S1. We stained with a Fixed Viability Dye (eBioscience) at 1:500 dilution in PBS per manufacturer's instructions. For surface staining, we incubated cells in flow cytometry buffer (PBS with 2% BSA and 2 mM EDTA) along with 50% Brilliant Stain Buffer (BD Horizon) per manufacturer's instructions. Surface antibodies included CD3 APC-R700, CD4 BUV395, CD25 BV786, CCR6 BV421, CCR4 PE-CF594 (BD Horizon); CD127 BV650, CD45RA APC-Cy7 (BioLegend); and CD45RO PerCP-eFluor710, CXCR3 PE-Cy7 (eBioscience). For intracellular staining, we used T-bet PE and RORγt Alexa Fluor 488 (BD Pharmingen) antibodies along with the Transcription Factor Buffer Set (BD Pharmingen) for both fixation and permeabilization. To set gating parameters for each fluorophore, we used fluorescence minus one (FMO) controls (38–41). We analyzed the same internal reference standard of PBMC for all batches to assess for staining variation with each batch acquisition. To standardize voltages across batches, we used Cytometer Setup and Tracking (CS&T) beads (BD Pharmingen) to measure median fluorescence intensities (MFI) and then matched voltages to these MFI values for each batch (42, 43). We used Ultracomp^TM^ beads (Invitrogen) as compensation controls with each batch. We collected at least 2 ×10^5^ events for each sample.

### Gating Strategy

We used FlowJo^TM^ software v10.0.07 (Becton, Dickinson and Company: Ashland, OR) to analyze the flow cytometry data. Compensation was performed using Ultracomp^TM^ beads. We employed our previous gating strategy to identify Th populations (11). Gating steps were as follows: (**1**) singlets using forward scatter (FSC—height vs. area), (**2**) live cells (negative for the fixed viability dye), (**3**) lymphocytes based on FSC and side scatter (SSC), (**4**) CD3^+^ cells, (**5**) CD4^+^ cells, (**6**) Non-T regulatory (NonTreg) cells defined as CD25^−^ and either CD127^Lo^ or CD127^Hi^, (**7**) T-effectors (CD45RA^−^ and CD45RO^+^), and (**8**) the Th17.0, Th17.1, and Th1 subsets were gated based on staining for CCR6, CCR4, and CXCR3 (Table 1). We first determined the RORγt and T-bet distributions among the three Th subsets. Because RORγt is the transcription factor that defines the Th17 lineage (13–17), our primary analyses were based on Th17.0 cells that expressed RORγt and we present data for the proportion of these cells that stained positive for T-bet based on FMO control samples.

**Table 1:** Definitions of Th subsets based on surface chemokine receptor expression.

	**CCR6**	**CXCR3**	**CCR4**
**Th17.0**	+	–	+
**Th17.1**	+	+	–
**Th1**	–	+	–

### Data Analysis

Data were analyzed in Stata/SE 15.1 software (StataCorp LLC: College Station, TX) and figures were constructed in GraphPad Prism 6 software (GraphPad Software, Inc.: La Jolla, CA). To compare demographics data, we used *t*-tests for two group comparisons of means from parametric data, analysis of variance analysis (ANOVA) for comparisons of means between three or more groups, and chi-squared testing to compare proportions between groups. We created several linear regression models where the T-bet^+^ frequency (%) of RORγt^+^Th17.0 cells was the dependent variable. In these models, we examined case status as a binary predictor with or without adjustment for confounders. In separate models, we compared those with PFT declines and PFT increases along with controls as distinct groups using a categorical predictor with or without adjustment for confounders. Where indicated, we adjusted our regression models for several confounders including age, sex, race, binary designations for immunosuppression use (yes/no), and prior smoking history (yes/no). In a subset of cases with PFT declines (these cases were not on immunosuppression) and PFT increases (these cases were either on or off immunosuppression), we used mixed effects linear regression models to determine if the T-bet^+^ frequency of RORγt^+^Th17.0 cells was associated with the direction of PFT change. The fixed effects were the PFT change designations as well as the clinical covariates (age, sex, race, immunosuppression use, and smoking history), and the random effects were the subjects. We specified unstructured covariation matrices in these mixed effects models (44, 45).

We used linear regression models to determine how the T-bet^+^ frequency of RORγt^+^Th17.0 cells varied based on the number of total organs involved, where thoracic adenopathy and/or lung parenchymal involvement was considered as one organ. These regression models had either binary (one or greater than one organ) or categorical (each organ number as its own group) designations for total organ number. For the categorical model, five or more organs was considered one group to ensure sufficient numbers of subjects in each category and we also performed a *post-hoc* linear trend test.

We used Pearson correlation analyses to determine the association between T-bet^+^ frequencies of RORγt^+^Th17.0 cells and previously measured blood markers related to IFN-γ, including serum levels of the IFN-γ-induced chemokines of CXCL9, CXCL10, and CXCL11 (34, 35) as well whole blood RNA transcript levels for IFN-γ-related genes (33). We also used linear regression models to adjust for potential confounders. We calculated correlation r values for these models by taking the square-root of the adjusted R^2^ from the linear regression models. For all regression models, we used robust standard errors (46, 47). We considered an α < 0.05 as significant and report two significant digits for all analyses.

## Results

### Characteristics of Sarcoidosis Subjects

We identified 33 subjects who met the PFT change threshold case definition (22 with declines and 11 with increases) and 10 subjects who met the case definition for control subjects; the clinical characteristics of these subjects are shown in Table 2 and details regarding their immunosuppression use are shown in Table S2. For the cases, the average time between visits where a PFT change occurred was 19 months (*SD* = 11). Cases who had PFT increases had lower values of DLCO and FVC at first measurement than the other groups (cases with declines or controls).

**Table 2:** Sarcoidosis characteristics at enrollment/time of blood draw in cases and controls.

	**Cases**		**Controls**	*p*-value^*^
	**PFT** **Decrease**	**PFT** **Increase**		
	***N* = 22**	***N* = 11**	***N* = 10**	
**Age (mean years, SD)**	56 (12)	50 (13)	46 (9)	0.082
**Female (%)**	16 (73)	4 (36)	3 (30)	0.089
**Race (%)**				0.37
African American	1 (5)	2 (18)	0 (0)	
White	19 (86)	7 (64)	8 (80)	
Hispanic	2 (9)	0 (0)	0 (0)	
Other	0 (0)	2 (18)	2 (20)	
**Ever Smokers**	11 (50)	6 (55)	4 (40)	0.64
**Immunosuppression Use (%)**	9 (41)	6 (55)	0 (0)	N/A
**Extra-thoracic Organ Involvement (%)**	75	78	10	0.00056
**Initial Visit PFTs: Mean (SD)**				
FVC %predicted	97 (16)	80 (18)	99 (13)	0.012
FEV1 %predicted	89 (18)	75 (24)	89 (17)	0.11
FEV1/FVC	0.72 (0.085)	0.72 (0.14)	0.71 (0.081)	0.95
DLCO %predicted	74 (16)	59 (13)	79 (12)	0.011
TLC %predicted	99 (11)	83 (17)	93 (13)	0.027

* *p-values are for comparisons between cases and controls*.

*p-values for PFTs compare the controls and the cases as two separate groups*.

*N/A, not applicable*.

### Transcription Factor Expression in Th Cells

**Figure 1:**
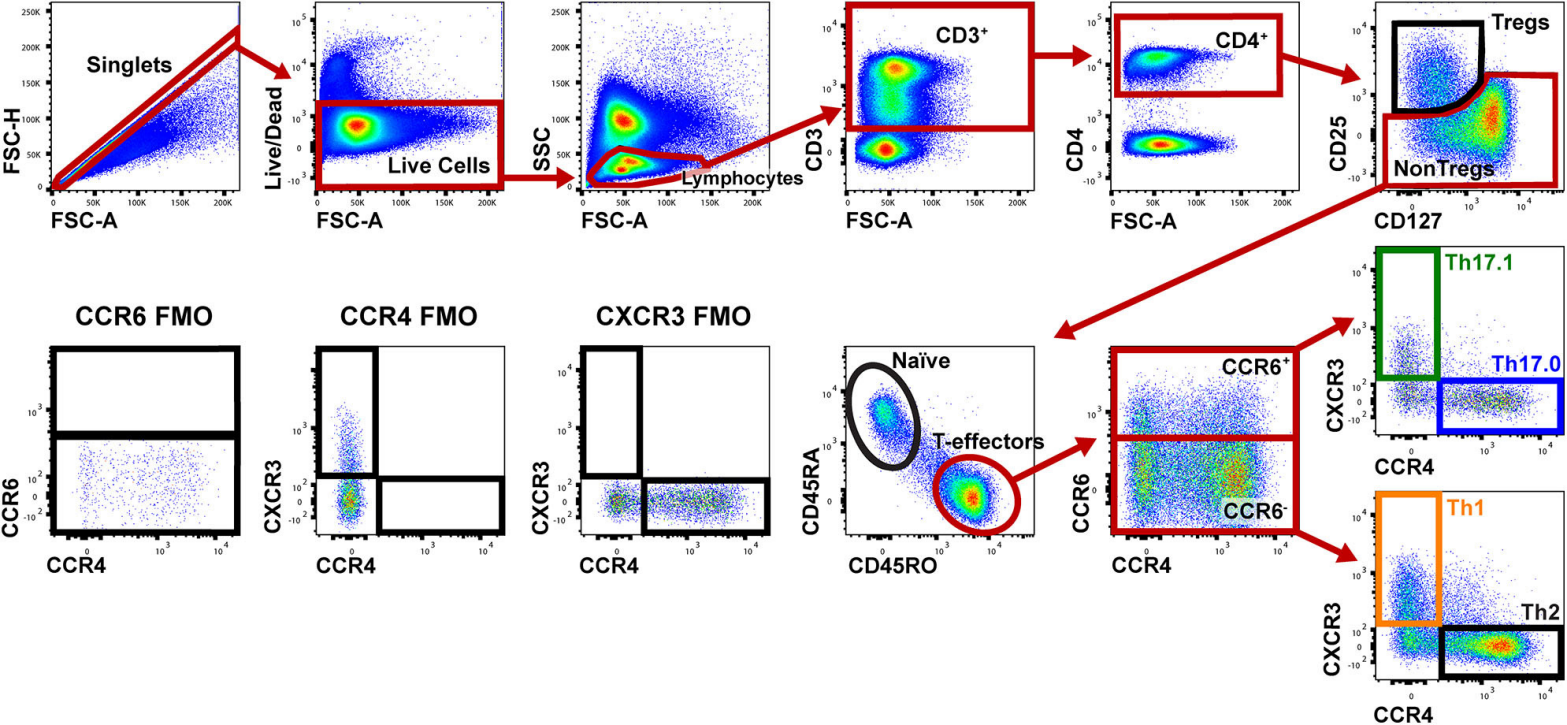
Gating strategy to identify Th populations among T-effector cells. Shown is a representative sarcoidosis subject sample. We gated on singlet cells using FSC-H and FSC-A, then live cells (negative for the fixed viability dye), lymphocytes based on FSC and SSC, then CD3^+^ and CD4^+^ cells. We then gated on NonTregs that were CD25^−^and either CD127^Lo^ or CD127^Hi^ and then T-effectors (CD45RA^−^ and CD45RO^+^). Among these T-effectors, we enriched for Th subsets using CCR6, CCR4, and CXCR3. Th17.0 cells were CCR6^+^CCR4^+^CXCR3^−^, Th17.1 cells were CCR4^−^CCR6^+^CXCR3^+^ and Th1 cells were CCR6^−^CCR4^−^CXCR3^+^. The gating strategy used for fluorescence minus one (FMO) controls for CCR6, CCR4, and CXCR3 are shown in the lower left corner.

**Figure 2:**
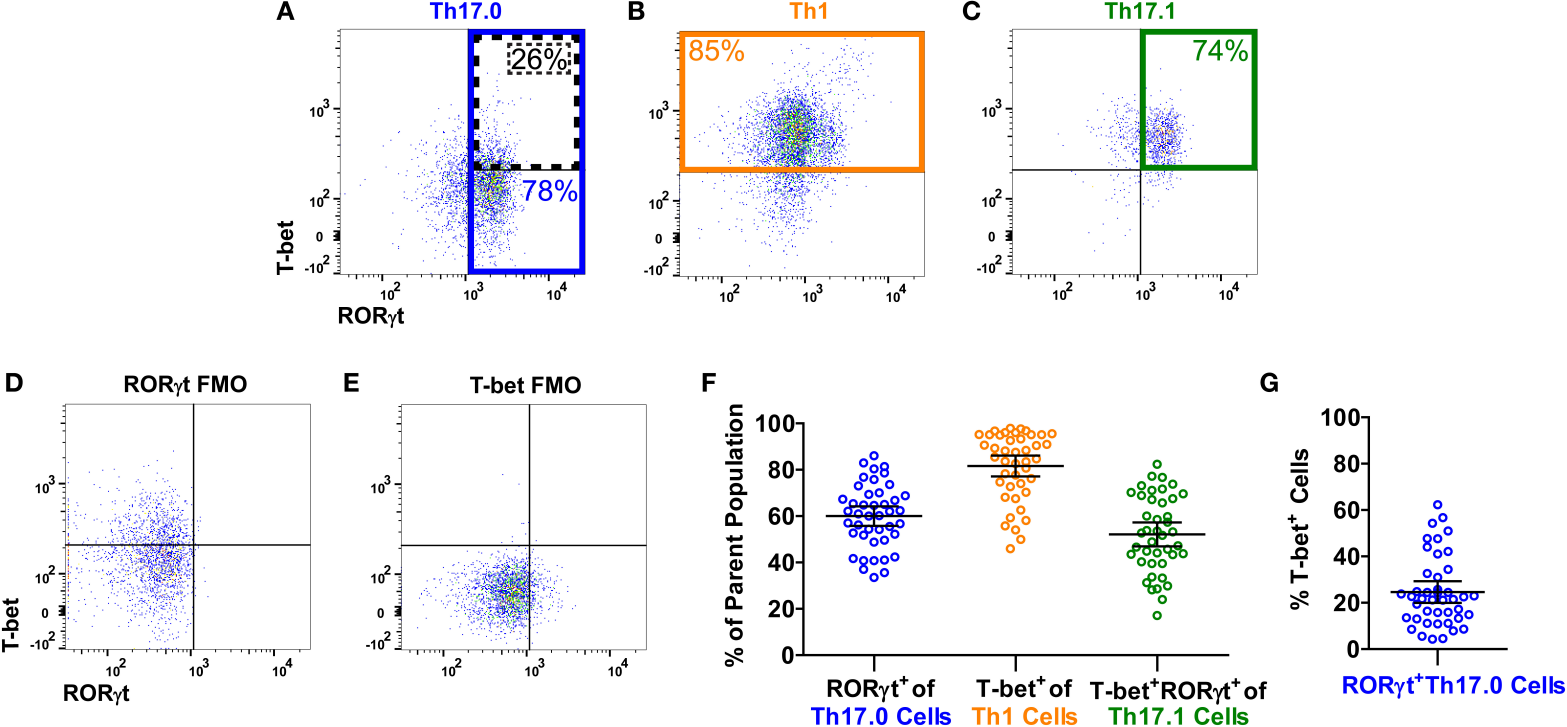
T-bet and RORγt expression in the Th17.0, Th1, and Th17.1 cell populations. For each Th population (as defined in Figure 1), we determined the expression of T-bet and RORγt, as shown in a representative sarcoidosis sample. Expression of T-bet and RORγt in **(A)** Th17.0, **(B)** Th1, and **(C)** Th17.1 cells along with fluorescence minus one controls (FMOs) for **(D)** RORγt and **(E)** T-bet are displayed as dot plots. The majority of cells in each Th population had the expected expression pattern of T-bet and RORγt based on its chemokine receptor pattern: **(A)** the majority of Th17.0 cells expressed RORγt (outlined in blue); **(B)** the majority of Th1 cells expressed T-bet (outlined in yellow); and **(C)** the majority of Th17.1 cells expressed both RORγt and T-bet (outlined in green). These frequencies of RORγt^+^ and/or T-bet^+^ cells in each of these Th cell populations for this representative subject sample are shown on each plot with corresponding colors. These frequencies are displayed graphically in **(F)** across all subjects where each open circle represents a single subject along with the mean and 95% confidence interval (CI). Our primary population of interest in this study was the Th17.0 subset, so to achieve the highest specificity for the “Th17.0” phenotype, we focused on RORγt^+^Th17.0 cells as outlined in blue in **(A)**. We found that some of these RORγt^+^Th17.0 cells also expressed T-bet, as outlined by the dotted black box in **(A)**. There was a range of T-bet^+^ cells within this RORγt^+^Th17.0 population across subjects, as shown graphically in **(G)**, where each open circle represents a subject along with the mean and 95% CI. Our subsequent analyses focused on how this T-bet^+^ frequency among RORγt^+^Th17.0 cells related to clinical sarcoidosis outcomes.

Figure 1 shows the gating strategy to identify the Th populations of interest (Th17.0, Th17.1, and Th1) among T-effectors cells based on surface expression of chemokine receptors. Because the polarization of each of these Th cell populations is influenced by the RORγt and T-bet transcription factors (16, 17, 26–28, 48, 49), we also measured the expression of these transcription factors as part of our staining panel (Figures 2A–C). We used FMO staining for RORγt and T-bet to set gates for positive and negative expressing cells as shown in Figures 2D,E (for FMO controls for each of our staining reagents, please refer to Figure S2). Figures 2A–C,F shows that using chemokine receptors to identify these Th cell subsets is sensitive but not as specific as also including transcription factors staining. As shown, the majority of Th17.0 cells express RORγt, most of the Th1 cells express T-bet, and the majority of Th17.1 cells express both RORγt and T-bet; further data to illustrate the concordance between these chemokine receptor patterns and transcription factor expression are shown in Figure S3. Although Tregs were not the focus of this study, we found that CD25^Hi^CD127^Lo^CD4^+^ T cells were present in the blood and represented 6.7% (95% CI 5.4–8.0) of total CD4^+^ T cells. Because the Th cell population of interest in this study was the Th17.0 subset, to increase the specificity of this population, we defined these cells by including expression of RORγt as shown by the solid blue box in Figure 2A and refer to them as RORγt^+^Th17.0 cells. We found that some of these RORγt^+^Th17.0 cells also expressed T-bet as shown in Figure 2A by the dashed black box. Figure 2G shows the range of T-bet expression in RORγt^+^Th17.0 cells across subjects. This T-bet^+^ frequency of RORγt^+^Th17.0 cells was the focus of our subsequent analyses.

### Relationship Between the T-bet^+^ Frequencies of RORγt^+^Th17.0 Cells and Clinical Outcomes

**Figure 3:**
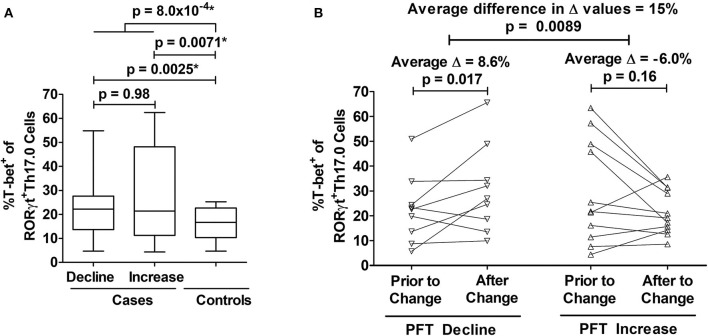
Associations between T-bet^+^ frequencies of RORγt^+^Th17.0 cells and pulmonary function changes. **(A)** Cases had higher %T-bet^+^ frequencies of RORγt^+^Th17.0 cells compared to controls in either adjusted or unadjusted models (*p-values from model adjusted for age, sex, race, immunosuppression use, and prior smoking). Cases were defined by declines (*n* = 22) or increases (*n* = 11) in either forced vital capacity (FVC) or diffusing capacity (DLCO) of 10 or 15%, respectively during follow up regardless of immunosuppression. Controls (*n* = 10) lacked these same pulmonary function test (PFT) changes and never required immunosuppressive treatment. The upper most *p*-value represents the result from a regression model that compared all cases to controls. The middle two *p*-values represent results from a regression model that distinguished cases as separate groups based on either PFT declines or increases and compared these groups to controls; the lower most *p*-value represents the results from this same regression model where cases with PFT declines were compared to cases with PFT increases. Data are displayed as box-and-whisker plots with median and interquartile ranges. **(B)** As assessed by mixed effects modeling adjusted for age, sex, race, immunosuppression use, and prior smoking, cases had either an increase (*n* = 9) (left panel) or decrease (*n* = 11) (right panel) in T-bet^+^ frequencies at the visit at which their PFT change occurred. The difference in the magnitude of these changes between those with PFT declines and PFT increases was 15%. In **(B)**, each subject's T-bet^+^ frequency is represented by an open symbol and are plotted based on when they were sampled relative to the PFT change.

We determined how the %T-bet^+^ frequencies of RORγt^+^Th17.0 cells varied between cases and controls. Cases had higher T-bet^+^ frequencies compared to controls (27 vs. 16%, *p* = 0.0040) in unadjusted analysis; this difference was also statistically significant in a model adjusted for age, sex, race, immunosuppression use, and prior smoking status as shown in Figure 3A. We also found that as separate groups, those with PFT declines and those with PFT increases had higher T-bet^+^ frequencies of RORγt^+^Th17.0 cells compared to controls in both unadjusted and adjusted models (Figure 3A). Of note, we did not find any association between the PFT groups and any of the T helper populations as defined by chemokine receptors and also using RORγt and/or T-bet to delineate these populations (Th17.0, Th17.1, Th1, or Th2) (see Table S3). We used a mixed effects model adjusted for age, race, sex, immunosuppression use, and prior smoking, to examine the T-bet^+^ frequencies of Th17.0 cells in relation to the direction of PFT change. We found that being in the PFT decline group was associated with an increase in T-bet^+^ frequency (average change = 8.6%, 95% CI 1.5–16, *p* = 0.017) (Figure 3B). Conversely, being in the PFT increase group was associated with a decrease in T-bet^+^ frequency (average change = −6.0%, 95% CI −14 to 2.4, *p* = 0.16) (Figure 3B). This model included an interaction term between the PFT change group (decline or increase) and the visit variable to assess if the change in T-bet^+^ frequency between the two visits differed based on the direction of PFT change. We found that there was a statistically significant interaction between the direction of PFT change and the visit variable. The magnitude of this difference was 15 percentage points (95% CI 3.7–26, *p* = 0.0089).

### Association Between T-bet^+^ Frequencies of RORγt^+^Th17.0 Cells and Organ Involvement

**Figure 4:**
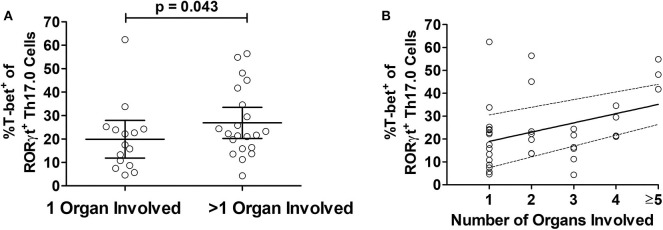
Relationship between T-bet^+^ frequencies of RORγt^+^Th17.0 cells and organ involvement. **(A)** The frequencies of %T-bet^+^ of RORγt^+^Th17.0 cells were higher in those with greater than one organ involved as compared to only one organ (*p*-value adjusted for age, sex, race, and immunosuppression use). **(B)** The T-bet^+^ frequencies of RORγt^+^Th17.0 cells where higher in those with greater number of organs involved. In a linear regression model with total organ involvement as a categorical predicator adjusted for age, sex, race, and immunosuppression use, there was a positive trend toward increasing T-bet^+^ frequencies of RORγt^+^Th17.0 cells with greater organ involvement (see Table 3). The solid line shows the organ number adjusted for age, sex, race, and immunosuppression use and the dashed lines represent the 95% confidence interval.

We considered the total number of organs involved with sarcoidosis at enrollment into the study as a separate manifestation of granulomatous disease burden. To test if T-bet expression in RORγt^+^Th17.0 cells varied based on the number of organs involved, we used linear regression models and adjusted for age, sex, race, and immunosuppression use. T-bet^+^ frequencies of RORγt^+^Th17.0 cells were 11 percentage points higher on average in those with more than one organ involved relative to those with a single organ involved (Table 3). We also constructed a model wherein the number of organs involved was a categorical predictor (from 1 organ to >5 organs) and found that the T-bet^+^ frequencies increased as the number of organs involved increased, especially with ≥ 5 organs involved (trend test, *p* < 0.001) (Table 3 and Figure 4).

**Table 3:** Linear regression using two different variables to delineate the number of involved organs.

**Outcome**	**Predictor**	**β-Coefficient**	**95% CI**	***p*-value**
%T-bet^+^ of RORγt^+^Th17.0 cells	Model (1) Binary: >1 Organ (1 Organ = Ref)	11%	(0.36, 22)	**0.043**
	Model (2) Categorical:		Trend Test:	**1.2 × 10**^−38^^‡^
	1 Organ	(Ref)	(Ref)	(Ref)
	2 Organs	11%	(−2.9, 24)	0.12
	3 Organs	3.5%	(−7, 14)	0.51
	4 Organs	9.4%	(−3.4, 22)	0.15
	≥5 Organs	25%	(16, 34)	**9.8** **×** **10**^**−8**^

† *Adjusted for age, sex, race, and immunosuppression use*.

‡ *p-value for the test of linear trend for the categorical organ variable*.

*Ref, Reference*.

### Correlations Between the T-bet^+^ Frequencies of RORγt^+^Th17.0 Cells and Other IFN-γ-Related Blood Markers

To assess if T-bet expression in RORγt^+^Th17.0 cells was associated with other markers of IFN-γ-related inflammation, we determined the correlations between these T-bet^+^ frequencies and serum levels of IFN-γ-induced chemokines and whole blood gene transcript levels of IFN-γ-related genes, which we had previously measured in the same blood samples (33–35). We found that the T-bet^+^ frequencies positively correlated with all three serum chemokines (CXCL9, CXCL10, and CXCL11) in models adjusted for age, race, sex, and immunosuppression use (Table 4). We previously found that a three gene mean of *GBP1, STAT1*, and *STAT2* was higher in a larger number of subjects from this cohort who had either PFT declines or flares requiring immunosuppression use (33). This three gene mean (the “IFN Factor”) as well as genes identified to be related to IFN-γ using Ingenuity Pathway Analysis (*TAP1* and *JAK2*) were positively correlated with the T-bet^+^ frequencies of RORγt^+^Th17.0 cells (Table 4).

**Table 4:** Results from correlation analyses and regression models for T-bet^+^ frequencies of RORγt^+^Th17.0 cells and IFN-γ-related blood markers.

**Outcome**	**Main Predictor**	**Adj β-coeff^†^**	**95% CI**	**Adj *p-*value**	**Adj r value**	**Unadj r value^‡^**
%T-bet^±^ of	Log_10_[CXCL9]^**§**^	22	(14, 31)	**1.5** **×** **10**^**−5**^	0.66	0.65
RORγt^±^Th17.0 cells	Log_10_[CXCL10]	23	(3.6, 42)	**0.022**	0.47	0.41
	Log_10_[CXCL11]	30	(11, 49)	**0.0028**	0.51	0.50
	IFN Factor**^††^**	6.9	(0.81, 13)	**0.028**	0.37	0.58
	*JAK2*^‡‡^	4.2	(2.5, 10)	**0.034**	0.29	0.42
	*TAP1*^‡‡^	4.8	(0.25, 9.3)	**0.041**	0.26	0.42

† *β-coefficient is adjusted for age, race, sex, and immunosuppression use*.

‡ *Unadjusted Pearson r coefficient*.

§ *Serum chemokine values were log_10_-transformed*.

†† *The “IFN Factor” = a three gene mean of GBP1, STAT1, and STAT2 previously measured from whole blood*.

‡‡ *Whole blood gene expression values in the form of log_2_[relative expression]*.

*Adj, Adjusted; CI, Confidence Interval; Coeff, Coefficient; Unadj, Unadjusted*.

*Bold values indicates p-values for adjusted β-coeffecients*.

## Discussion

Sarcoidosis is a systemic disease involving granulomatous inflammation with upregulation of immune pathways related to IFN-γ (5, 6, 50, 51). In light of recent findings that IFN-γ-producing Th17.1 cells are elevated in the lungs and lymph nodes of sarcoidosis patients with chronic disease (52), the ontogeny and function of these cells may be important in the pathogenesis of sarcoidosis (11, 52). This study was motivated by (1) clinical observations that Th17.0 cells are elevated in blood while Th17.1 cells are elevated in BAL fluid and mediastinal lymph nodes of sarcoidosis patients (11, 52), and (2) scientific evidence from mice and humans demonstrating the plasticity of Th17.0 cells to become Th17.1 cells after exposure to IL-12 and IFN-γ and upregulation of T-bet (21–24). Since granulomas are a source of IL-12 and IFN-γ (53, 54), we hypothesized that T-bet expression in peripheral Th17.0 cells would reflect the extent of granulomatous inflammation in sarcoidosis patients. Therefore, we used transcription factor staining for T-bet and RORγt along with chemokine receptor staining to identify Th17 cell populations, specifically T-bet^+^ frequencies of RORγt^+^Th17.0 cells. We found that these T-bet^+^ frequencies were higher in sarcoidosis subjects with clinical evidence of greater disease burden as manifested by clinically meaningful PFTs changes and organ involvement.

Given the limitations in directly quantifying the degree of inflammation present in our human subjects, our study design utilized clinical features of disease severity as indicators of the extent of granulomatous inflammation. Specifically, we used a case-control study design to compare sarcoidosis subjects with different clinical courses. We assumed that those with any type of PFT change (our cases) had greater amounts of granulomatous inflammation during the study period as compared to those who had stable PFTs and did not require immunosuppression (our controls). The greater T-bet^+^ frequencies of RORγt^+^Th17.0 cells in our cases supports our hypothesis that this T-bet^+^ frequency measure reflects the extent of disease burden. In subgroup analyses of our cases, we found that the T-bet^+^ frequencies of RORγt^+^Th17.0 cells were increased following a PFT decline. Conversely, T-bet^+^ frequencies were decreased following a PFT improvement. We interpret these findings as evidence that these T-bet^+^ frequencies changed based on disease trajectory and therefore varied based on the extent of granulomatous inflammation over time. We also used the number of organs involved as another indicator of disease burden. Thus, we interpreted the positive association between these T-bet^+^ frequencies and organ involvement as further evidence that these T-bet^+^ frequencies were associated with the extent of granulomatous inflammation. As additional support for our findings, we found positive correlations between these T-bet^+^ frequencies and other measures of IFN-γ-related inflammation, including serum interferon-induced chemokines and whole blood gene transcript levels.

The polarization of Th17.0 cells to Th17.1 cells has been studied both in mice and several human diseases including rheumatoid arthritis, multiple sclerosis, and Crohn's disease (12, 17, 55–62). Mechanisms currently put forth for how Th17.0 can become Th17.1 involve upregulation of T-bet. It has long been established that T-bet expression in naïve T cells leads to the acquisition of a Th1 phenotype after antigenic stimulation in the presence of cytokines such as IL-12 and resulting upregulation of STAT1 (25–29). This process includes T-bet's role in the upregulation of CXCR3 expression, which is essential for trafficking of Th1 cells to sites of inflammation (26, 27, 48). During and post-polarization, T-bet also upregulates IFN-γ through binding to both the promoter and enhancer loci for *IFNG* (29). Mouse models have shown that Th17.0 cells incubated with IFN-γ and IL-12, or TNF-α gain features of Th17.1 cells including CXCR3 expression and the capacity to produce IFN-γ (22–24). This phenomenon of plasticity from IL-17-producing cells to IFN-γ-producing cells has also been observed in mouse models of innate lymphoid cells, where RORγt^+^ ILC3 cells transition to ILC1 cells through upregulation of T-bet in the presence of specific cytokines such as IL-12 (63–66). A human study of inflammatory bowel disease found that *ex vivo* IL-12 stimulation led to IFN-γ production in Th17 cells that were isolated as CCR6^+^CXCR3^−^ cells from mesenteric lymph nodes (17). This observation suggests that upregulation of CXCR3 may occur later in the cellular differentiation of Th17.0 to Th17.1 cell phenotype. Other groups including Cohen et al. (21) showed that polarized Th17.0 cells derived from *ex vivo* human PBMCs could subsequently upregulate the T-bet gene *TBX21* after incubation with IL-12 and IFN-γ. Therefore, T-bet expression in RORγt^+^Th17.0 cells (CCR6^+^CXCR3^−^CCR4^+^) could potentially represent a transitional state between the Th17 and Th17.1 immune subsets. Our focus on Th17.0 cells (and not Th17.1 cells that also express CXCR3), was motivated by our goal to identify cells prior to upregulation of CXCR3, since expression of this chemokine receptor could lead to trafficking of cells out of the blood.

Where the transition between Th17.0 and Th17.1 occurs *in vivo* has been an on-going question in studies of T cell biology. In the setting of other granulomatous processes such as pulmonary tuberculosis infection, mouse models have shown that dendritic cells present antigen to naïve T cells in mediastinal lymph nodes (67–69). Therefore, the initial polarization of Th1 cells in this disease model does not occur in the alveoli, but instead these polarized cells must traffic to the initial site of infection after their activation in local lymph nodes. For Th17.0 cells, mouse models studying gastrointestinal T cells have shown that Th17.0 cells initially become polarized in the mesenteric lymph nodes then traffic through the blood to the intestine in models of both healthy and inflammatory states (70, 71). The location of Th17.1 polarization potentially includes both lymph nodes and inflamed tissue. In a mouse model of experimental autoimmune encephalitis, single-cell RNA sequencing analysis showed that Th17 cells isolated from lymph nodes and affected central nervous system tissues have several phenotypes with respect to cytokine production (62). These phenotypes ranged from self-renewing IL-17-producing cells and IFN-γ-producing cells in the lymph nodes to IFN-γ-producing cells in inflamed tissues. In a mouse model of colitis, Harbor et al. (59) showed that naïve T cells polarized to Th17.0 cells *ex vivo* and then injected into the peritoneum were later retrieved and found to be competent to produce IFN-γ. These cells were recovered from both mesenteric lymph nodes and inflamed intestinal tissue, suggesting that Th17.0 cells could be subsequently polarized to Th17.1 cells in either compartment. Taken together, these findings suggest that the transition from Th17.0 to Th17.1 cell may occur in both lymph nodes and inflamed tissues.

These mouse and human studies of Th17 plasticity provide a framework for how the transition from Th17.0 to Th17.1 cells might be occurring in sarcoidosis. Although it is unclear *where* the transition is occurring, we speculate that Th17.0 cells exposed to granuloma-related cytokines (i.e., IFN-γ, IL-12 and TNF-α) in the lymph nodes of affected organs, especially mediastinal lymph nodes, could lead to T-bet upregulation. We theorize that we are detecting this early T-bet^+^ transition state of Th17.0 cells in the blood as these cells exit the lymph nodes and enter the peripheral circulation. Eventually, increased T-bet expression in these Th17.0 cells results in upregulation of CXCR3, which is a key surface marker for the Th17.1 phenotype. Since CXCR3 is a homing receptor, its expression in Th17.1 cells leads to their accumulation in affected tissues. This conceptual framework is supported by several prior and current observations. Previously, we observed elevated frequencies of Th17.0 cells in the blood associated with elevated frequencies of Th17.1 cells in BAL (11). In the current study we observed a positive correlation between T-bet^+^ frequency in RORγt^+^Th17.0 cells and several IFN-γ-related blood markers including serum IFN-induced chemokines and gene transcript levels. These observations along with the associations of T-bet^+^ frequency in RORγt^+^Th17.0 cells with clinical outcomes lead us to infer that there is a systemic up-regulation of IFN-γ-related pathways and the degree to which these pathways are upregulated may be related to the burden of granulomatous inflammation in the body. Since these responses can be measured in the blood, they could be used to prognosticate patients as well as further our understanding of the underlying immunopathology.

Our findings share commonalities and differences with a prior publication of RORγt and T-bet expression in sarcoidosis subjects. Kaiser *et al*., showed strong associations between dual expression of CCR6 and CXCR3 in RORγt^+^T-bet^+^ T cells (10); we found similar strong associations between CCR6^+^ and CXCR3^+^ co-expressing cells we defined as Th17.1 cells that also had dual expression of RORγt and T-bet (see Figure 2C and Figure S3). However, our study differed in that we did not have BAL specimens and only focused on blood cells. The Kaiser study found that co-expression of RORγt and T-bet in CD4^+^ cells was higher in the BAL of patients with Löfgren syndrome as compared to non-Löfgren sarcoidosis, suggesting that co-expression predicted a more favorable phenotype. Our study differed in that we did not have patients with Löfgren syndrome, and we used longitudinal PFT changes, immunosuppression use, and total number of organs involved to phenotype subjects. The other difference was that our main focus was on T-bet-expressing RORγt^+^Th17.0 cells that did not express CXCR3 in order to identify potential transition cells in the blood, which was not addressed in the Kaiser study.

Some of the limitations of our study included our inability to directly assess the potential pathogenicity and IFN-γ-producing capabilities of the RORγt^+^Th17.0 cells that also expressed T-bet. Staining for transcription factors involves fixation and permeabilization of the cells, which prevents analyses involving stimulation and cytokine measurements after sorting these cells. Nonetheless, the association of the T-bet^+^ frequencies with clinically meaningful outcomes of sarcoidosis indicates that this cell population may be important to examine in future studies that further define the Th17.0 to Th17.1 transition. Our study was also limited by the lack of serially measured organ involvement and chest radiography, which precluded us from including these clinical findings in our definitions of sarcoidosis outcomes. Another important limitation relates to generalizability since our cohort was heterogeneous, and therefore our study design did not allow us to extrapolate the prognostic value of T-bet^+^ frequencies of RORγt^+^Th17.0 cells at initial diagnosis. Similarly, this study also was not designed to address the question of whether T-bet expression in RORγt^+^Th17.0 cells can predict the likelihood of spontaneous remission. Some of these limitations can be addressed in future studies that enroll subjects at the time of diagnosis. In terms of our study design, we created case and control definitions to maximize the likelihood of identifying those with active and inactive granulomatous inflammation. The limitation of doing this is that we used lack of immunosuppression use to help identify those who were more likely to have inactive disease, therefore use of immunosuppression was not matched between cases and controls. We dealt with this by including immunosuppression as a covariate in all our regression models to control for possible bias in our point estimates. Moreover, we would assume that immunosuppression use would decrease T-bet expression in controls even further, therefore the results we report here are likely more conservative than if we matched based on immunosuppression use.

## Conclusions and Future Directions

In summary, we provide evidence showing that T-bet expression in RORγt^+^Th17.0 cells was associated with both pulmonary and systemic organ involvement outcomes. These associations were potentially due to the effects of IFN-γ and IL-12 on Th17.0 cells as they circulate through affected lymph nodes where higher levels of these cytokines might be found. Future goals include determining how these frequencies in the blood relate to those in the lung, how they are associated with outcomes when measured at time of diagnosis, and how they change in response to treatment. With this information, we may be able to leverage biological data taken at the time of sarcoidosis diagnosis to inform patient prognosis and guide clinical decision making.

## Data Availability Statement

The raw data supporting the conclusions of this article will be made available by the authors, without undue reservation, to any qualified researcher.

## Ethics Statement

The studies involving human participants were reviewed and approved by University of California, San Francisco Institutional Review Board. The patients/participants provided their written informed consent to participate in this study.

## Author Contributions

NA and SM conducted the experiments. NA, IA, PW, and LK analyzed the data. All authors contributed to the writing and editing of the manuscript. All authors read and approved the final manuscript.

## Conflict of Interest

The authors declare that the research was conducted in the absence of any commercial or financial relationships that could be construed as a potential conflict of interest.

## Abbreviations

ANOVA: analysis of variance
BSA: bovine serum albumin
CI: confidence interval
CS&T: cytometer setup and tracking
DLCO: diffusing capacity of the lungs for carbon monoxide
DMARD: disease modifying antirheumatic drug
DMSO: dimethyl sulfoxide
EDTA: ethylenediaminetetraacetic acid
FBS: fetal bovine serum
FMO: fluorescence minus one
FEV1: forced expiratory volume in 1 s
FVC: forced vital capacity
FEV1/FVC: forced expiratory volume in 1 S to Forced vital capacity ratio
FSC: forward scatter
IFN-γ: interferon-gamma
IFN factor: interferon factor
MFI: median fluorescence intensity
NonTreg: Non-T regulatory
PBMC: peripheral blood mononuclear cell
%pred: percent predicted
RPMI: roswell park memorial institute
SD: standard deviation
SSC: side scatter
Th: T helper
TLC: total lung capacity
TNF-α: tumor necrosis factor alpha.
