# Supplementary material for: T-bet Expression in Peripheral Th17.0 Cells Is Associated With Pulmonary Function Changes in Sarcoidosis
Source: Front Immunol. 2020 Jul 22;11:1129. doi: 10.3389/fimmu.2020.01129 (PMC7387715; doi:10.3389/fimmu.2020.01129)
Supplement: Supplementary file 1 [file Data_Sheet_1.docx]

**Supplementary Material**


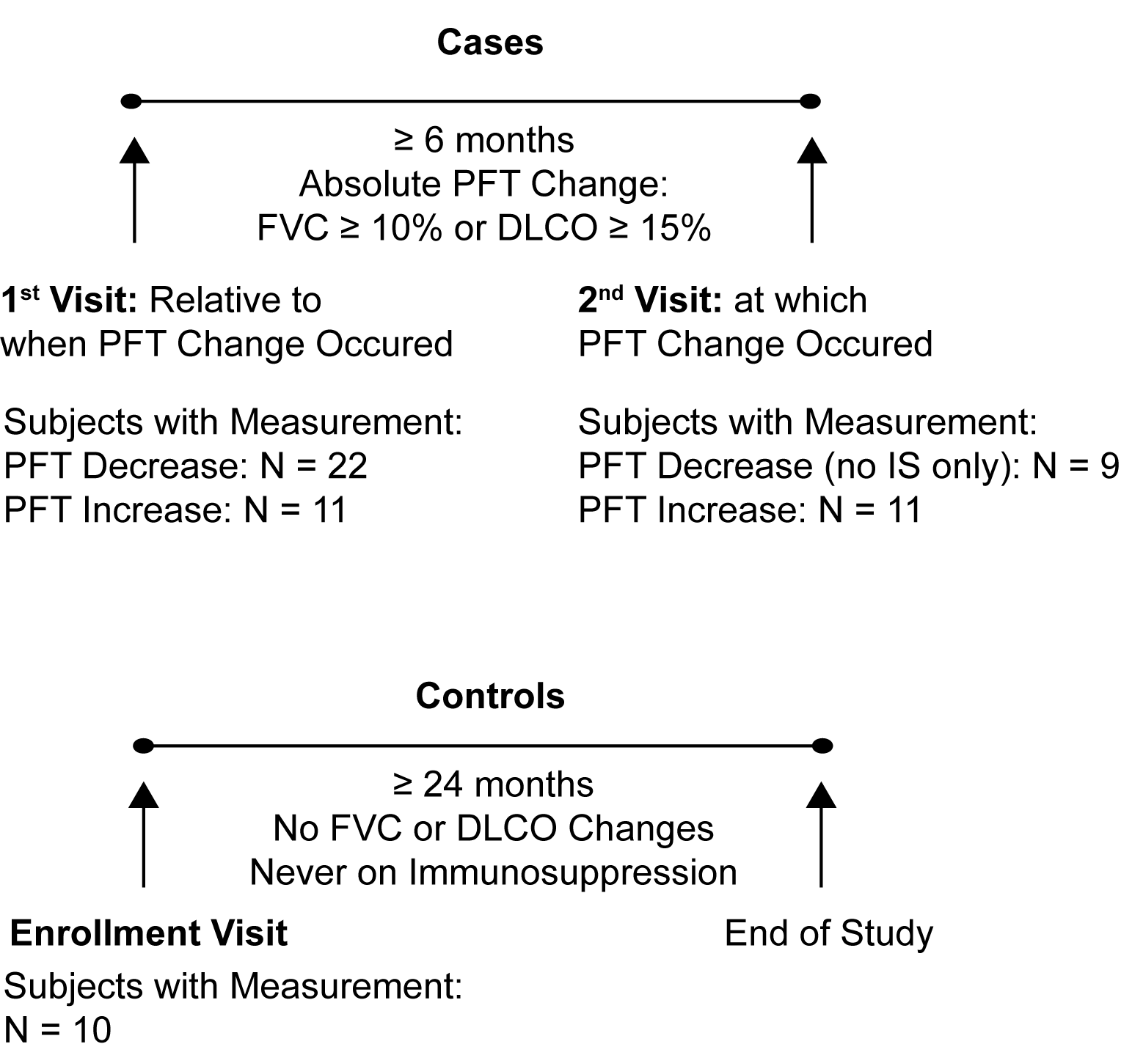


**Supplementary Figure S1.** Diagram of case-control design. Cases and controls were chosen based on meeting specific criteria as depicted. Abbreviations: IS = immunosuppression.

| **Supplementary Table S1.** Antibody staining reagents | | | | |
| --- | --- | --- | --- | --- |
| **Antigen/Stain** | | **Conjugate** | **Clone** | **Company** |
| Fixed Viability Stain | eFluor506 | |  | eBioscience |
| CD3 | APC-R700 | | UCHT1 | BD Horizon |
| CD4 | BUV395 | | RPA-T4 | BD Horizon |
| CD25 | BV786 | | M-A251 | BD Horizon |
| CD127 | BV650 | | A019D5 | BioLegend |
| CD45A | APC-Cy7 | | HI100 | BioLegend |
| CD45RO | PerCP-eFluor710 | | UCHL1 | eBioscience |
| CCR4 | PE-CF594 | | 1G1 | BD Horizon |
| CCR6 | BV421 | | 11A9 | BD Horizon |
| CXCR3 | PE-Cy7 | | CEW33D | eBioscience |
| RORγt | Alexa Fluor 488 | | Q21-559 | BD Pharmingen |
| T‑bet | PE | | O4-46 | BD Pharmingen |

| **Supplementary Table S2.**  Immunosuppressive medications used. | | | | | |
| --- | --- | --- | --- | --- | --- |
|  | **Cases** | | | | **Controls** |
|  | **PFT Decrease** | | **PFT Increase** | |  |
|  | N = 22 | | N = 11 | | N = 10 |
|  | **N (%)** | **Average Dose** | **N (%)** | **Average Dose** | **N (%)** |
| **Any immunosuppression** | 9 (41) |  | 6 (55) |  | 0 |
| 1 Medication | 9 |  | 4 |  |  |
| 2 Medications | 0 |  | 2 |  |  |
| **Prednisone** | 3 (14) | 43 mg/day | 2 (18) | 23 mg/day | 0 |
| **Methotrexate** | 3 (14) | 14 mg/week | 4 (36) | 14 mg/week | 0 |
| **Azathioprine** | 0 (0) |  | 0 (0) |  | 0 |
| **Infliximab** | 0 (0) |  | 1 (9.1) | 5 mg/kg every 2 wk | 0 |
| **Colchicine** | 1 (4.5) | 1.2 mg/day | 0 (0) |  | 0 |
| **Hydroxychloroquine** | 2 (9.1) | 400 mg/day | 1 (9.1) | 400 mg/day | 0 |


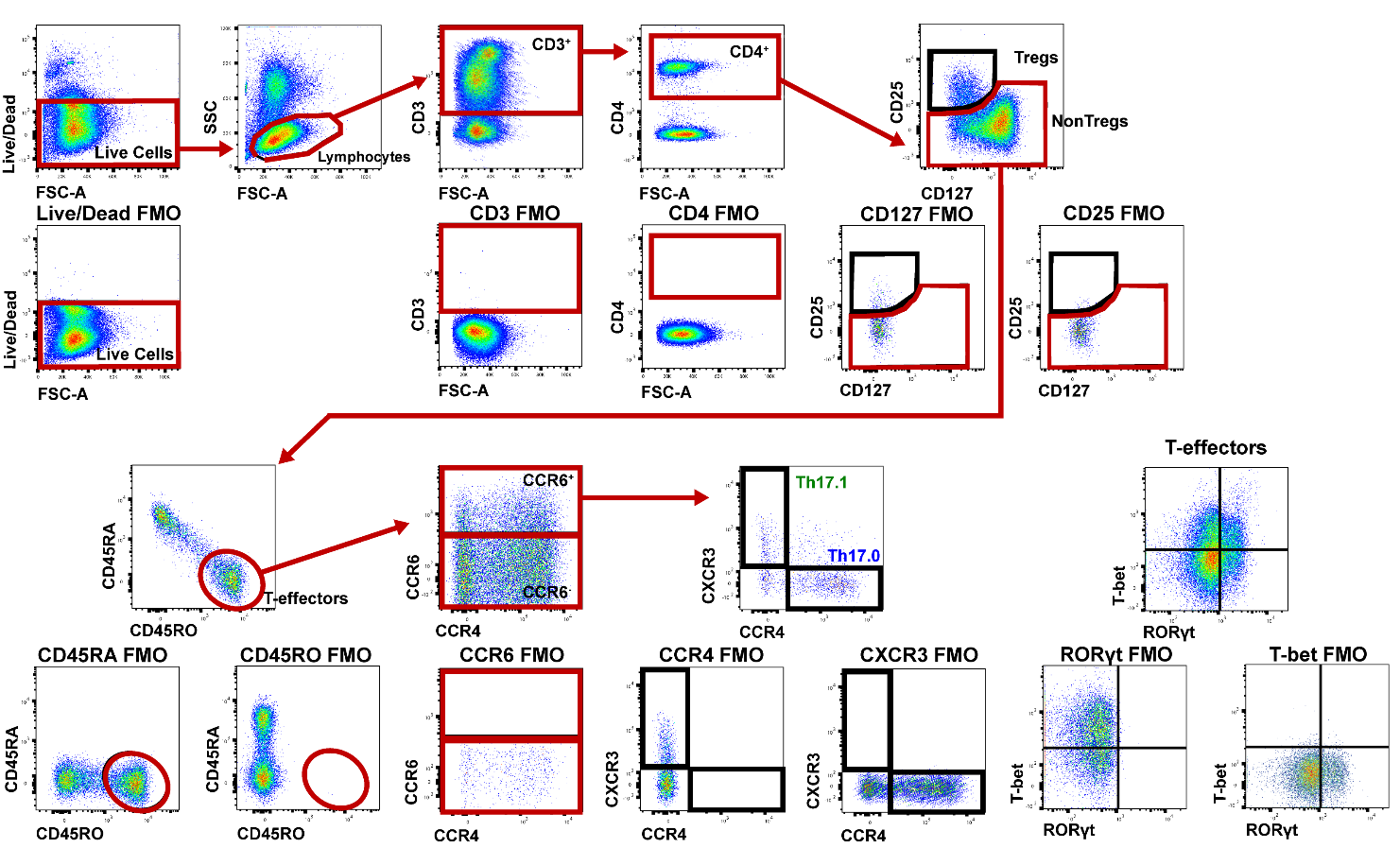


**Supplementary Figure S2.** Gating strategy with fluorescence minus one controls. For each staining marker, a fluorescence minus one (FMO) control was used to set positive and negative gates. For the Th populations, only CCR6^+^ populations are shown (see Figure 1 for full gating of these populations). T-bet and RORγt is shown for T-effectors; for RORγt and T‑bet expression in each Th subset, refer to Figure 2.





**Supplementary Figure S3.** RORγt and T-bet expression in the Th17.0, Th1, and Th17.1 populations as defined by surface chemokine receptors. The majority of cells in each Th population had the expected expression pattern of T-bet and RORγt based on its chemokine receptor pattern: **(A)** the majority of Th17.0 and Th17.1 cells expressed RORγt; **(B)** the majority of Th1 and Th17.1 cells expressed T-bet; and **(C)** the majority of Th17.1 cells expressed both RORγt and T‑bet, more so than either Th17.0 or Th1 cells. In each graph, data for all subjects are shown for each Th population where each open circle represents a single subject along with the mean and 95% confidence interval (CI).

| **Supplementary Table S3.** | | | | | | | | | |
| --- | --- | --- | --- | --- | --- | --- | --- | --- | --- |
| T helper populations frequencies based on case-control status. | | | | | | | | | |
| **T helper population** | **% of T-effectors**^†^ | | | | | | | | |
|  | 1. **Cases vs. Controls^††^** | | | 1. **Cases (PFT Decrease, PFT Increase) vs. Controls** | | | | | |
|  | **Cases** | **Controls** |  | **Cases** | | | | **Controls** | |
|  |  |  | **p-value** | **PFT Decline** | **p-value**  **(vs. Controls)** | **PFT Increase** | **p-value**  **(vs. Controls)** |  | |
| RORγt^+^Th17.0 cells^‡^ | 5.2 | 5.1 | 0.91 | 4.4 | 0.64 | 5.8 | 0.52 | 4.9 | |
| RORγt^+^T-bet^+^Th17.1 cells | 2.4 | 2.5 | 0.77 | 2.2 | 0.75 | 2.2 | 0.83 | 2.5 |  |
| T-bet^+^Th1 cells | 11 | 15 | 0.42 | 13 | 0.64 | 10 | 0.28 | 16 | |
| RORγt^-^T-bet^-^Th2 cells | 29 | 35 | 0.32 | 27 | 0.26 | 31 | 0.59 | 35 | |
| ^†^Average T helper frequencies as a percentage of T-effectors as calculated from regression models after adjusting for age, sex, race, immunosuppression, and prior smoking for 1) cases vs. controls or 2) cases (separated as PFT declines and PFT increases) vs. controls. For each Th subset, the difference between those with PFT declines and PFT increases also had p-value >0.05.  **^††^**Cases (PFT declines and increases) and controls (stable PFTs and never on immunosuppression)  ^‡^Populations defined by chemokine receptor and transcription factor expression. | | | | | | | | | |
